# Systematic review of safety and tolerability of a complex micronutrient formula used in mental health

**DOI:** 10.1186/1471-244X-11-62

**Published:** 2011-04-18

**Authors:** J Steven A Simpson, Susan G Crawford, Estelle T Goldstein, Catherine Field, Ellen Burgess, Bonnie J Kaplan

**Affiliations:** 1 Department of Psychiatry and Department of Oncology, University of Calgary, Calgary, Alberta, Canada; 2 Behavioural Research Unit, Alberta Children's Hospital, Calgary, Alberta, Canada; 3 San Diego, California, USA; 4 Department of Agricultural, Food and Nutritional Science, University of Alberta, Edmonton, Alberta, Canada; 5 Department of Medicine, University of Calgary, and Foothills Medical Center, Calgary, Alberta, Canada; 6 Department of Pediatrics and Department of Community Health Sciences, University of Calgary, Calgary, Alberta, Canada; 7 Behavioural Research Unit, Alberta Children's Hospital, 2888 Shaganappi Trail NW, Calgary, AB T3B 6A8 Canada

## Abstract

**Background:**

Theoretically, consumption of complex, multinutrient formulations of vitamins and minerals should be safe, as most preparations contain primarily the nutrients that have been in the human diet for millennia, and at safe levels as defined by the Dietary Reference Intakes. However, the safety profile of commercial formulae may differ from foods because of the amounts and combinations of nutrients they contain. As these complex formulae are being studied and used clinically with increasing frequency, there is a need for direct evaluation of safety and tolerability.

**Methods:**

All known safety and tolerability data collected on one complex nutrient formula was compiled and evaluated.

**Results:**

Data were assembled from all the known published and unpublished studies for the complex formula with the largest amount of published research in mental health. Biological safety data from 144 children and adults were available from six sources: there were no occurrences of clinically meaningful negative outcomes/effects or abnormal blood tests that could be attributed to toxicity. Adverse event (AE) information from 157 children and adults was available from six studies employing the current version of this formula, and only minor, transitory reports of headache and nausea emerged. Only one of the studies permitted a direct comparison between micronutrient treatment and medication: none of the 88 pediatric and adult participants had any clinically meaningful abnormal laboratory values, but tolerability data in the group treated with micronutrients revealed significantly fewer AEs and less weight gain.

**Conclusions:**

This compilation of safety and tolerability data is reassuring with respect to the broad spectrum approach that employs complex nutrient formulae as a primary treatment.

## Background

Nutrition guidelines need to be modified from time to time to remain current with research findings, but revisions are generated by sluggish processes involving scientific and governmental committees. The mismatch between the current speed of research on the health effects of various nutrients and the speed of guideline modification leaves health professionals and the public with imperfect information for making decisions about the safety of incorporating micronutrients into a treatment plan. This problem becomes more complex when one considers the 'non-healthy population,' as population guidelines were not developed to include these individuals. Nowhere is this challenge greater than with formulae containing more than one nutrient (complex nutrient formulae). Some believe that the strongest verification that micronutrient combinations are safe is the evidence from thousands of years of human food habits, as most preparations are primarily nutrients that have been in the human diet for millennia; however, their amounts and combinations differ from the way the nutrients occur in food. Consequently, safety and tolerability information on formulae that combine various micronutrients (vitamins, minerals, amino acids, essential fatty acids) could have potential value for many people who suffer from illnesses for which there are currently no (or severely limited) effective cures.

The Dietary Reference Intakes in North America (DRIs) provide guidance on the quantities of vitamins and minerals thought to be safe for long term ingestion by the healthy population, called the Tolerable Upper Intake Levels (ULs). But the DRIs pertain only to single-nutrient consumption, and their application to composite formulae can result in peculiar interpretations. For instance, the UL for folate is 1 mg/day because a higher level might mask a B12 deficiency. This effect may be a concern, but likely only to a specific group in the population at risk of B12 deficiency and only when an individual takes a single nutrient supplement. Most complex formulae with B vitamins would contain both, so the risk of masking a vitamin deficiency would be minimized. In addition, the DRIs and ULs are based on the healthy population and their application to individuals with a clinical diagnosis is not known. As there is an incomplete understanding of the nutrient needs of those not considered to be 'healthy' North Americans, by default the DRIs become the guidelines for everyone. Knowing the safety profile of a complex formula used by people with mental health diagnoses would add value beyond DRI information.

The potential unsuitability of applying the upper limits of the DRIs to multi-ingredient formulae aimed at those who might fall outside the definition of the 'healthy population' is important because the study of the health benefits of micronutrients has increased rapidly in the past decade. Various mixed or single nutrient formulae have been shown to increase resistance to communicable diseases [1], decrease the risk of birth defects [2], be effective in treating specific problems such as sexual dysfunction [3], prevent hip fractures [4], and improve immune function [5]. A randomized controlled trial (RCT) in 445 hospitalized elderly patients revealed significantly fewer re-admissions in those who received a broad-based vitamin-mineral treatment [6]. Recently, Shea and colleagues have been reporting positive benefits from a six-ingredient formula in patients with Alzheimer's [7,8]. Positive findings such as these increase the likelihood that research and clinical use of complex micronutrient formulae will continue to expand in the coming years. Hence, information on safety and tolerability is important for public health.

### Rationale

To further establish the relevance of the safety of multi-ingredient formulae to psychiatry, it is useful to address the available evidence on efficacy. Several RCTs of multi-ingredient formulae have demonstrated an impact on psychiatric symptoms such as antisocial and violent behavior. Schoenthaler reported a 28% decrease in rule violations in 62 imprisoned delinquents given a daily micronutrient formulation when compared to those who received a placebo [9]. Research on delinquent behavior in 80 schoolchildren aged 6-12 yielded similar results [10]: those receiving a complex micronutrient formula had a 53% lower rate of antisocial behavior requiring discipline (average 1/child) than the placebo group (average 1.875/child). In an RCT often erroneously cited as an investigation of a single ingredient (essential fatty acids; EFAs), there was a 35.1% decrease in disciplinary incidents (from 16 to 10.4 per thousand person-days) for 231 young offenders receiving a formula with 25 vitamins and minerals plus some EFAs, compared with a reduction of only 6.7% in those receiving a placebo [11]. Using a similar 26-ingredient formula in 221 young offenders, Zaalberg and colleagues partially replicated the results, finding 34% fewer reported prison 'incidents' for the group receiving the active formula, and a 14% increase in those who were taking the placebo [12].

In a study of 225 hospitalized elderly patients suffering from various acute illnesses [13], those receiving a complex micronutrient formula displayed fewer signs of depression on a 15-item geriatric depression scale than those receiving placebo, regardless of whether they had been clinically depressed. In other words, there was evidence of improved mood in everyone receiving the micronutrients, those with severe or mild depression, as well as others. A nonclinical sample of adults given a complex formula exhibited significant improvement on all psychometric measures of stress during a 30-day placebo-controlled trial [14]. In other research, decreases in anxiety and perceived stress were found in 80 normal healthy men who consumed a complex micronutrient formula compared to a placebo control [15].

Benton's extensive review article on nutrition and behavior covered both EFAs and other micronutrients [16]. In children with ADHD, there was no clear evidence of benefit from EFAs alone. In contrast, the studies that combined EFAs with vitamins and minerals (albeit in samples of young offenders) reported beneficial effects [11,12]. The reason multi-nutrient formulae demonstrate benefits may in part be due to underlying dietary inadequacy, but the results of the above studies where sometimes nutrients were given in relatively high amounts suggest that the mechanisms are more complex and likely relate to some of the underlying etiology or pathophysiology of psychiatric disorders.

With two exceptions, each of the studies mentioned above has used a unique combination of ingredients. One exception is the work of Shea and colleagues [7,8] who have reported more than one study using a single formula in various geriatric samples; the other is the study by Zaalberg [12] which used a very similar formula to the one studied by Gesch [11]. The formulae have varied considerably across studies, consisting of anywhere from three micronutrients to over 20.

There is only one complex micronutrient formula, EMPowerplus (EMP+), which has been studied extensively in mental health, by several research teams. The purpose of this paper is to provide safety and tolerability information on EMP+. In terms of PICOS (participants, interventions, comparisons, outcomes, and study designs), the review was all-inclusive and excluded no known source of data on this formula. Based on the theoretical issues mentioned above, there is reason to predict that this formula will not result in nutrient-related complications. However, it contains 36 ingredients, in a combination that likely does not occur naturally in the habitual intake of North Americans. Prior to any conclusions on potential efficacy, empirical data on its safety is essential for evaluating its potential for harm. With respect to the DRIs, there are four ingredients that exceed the ULs (cf. Table 1 for dose details and comparison to ULs), but none exceeds the Lowest Observed Adverse Event Level, and as explained in Table 1, surpassing the ULs for those four nutrients appears to be of no concern with respect to a complex formula's safety.

**Table 1:** Comparison of EMPowerplus ingredients with Tolerable Upper Intake Levels (ULs)

	Amount in a typical therapeutic dose, 15 capsules daily	UL
**Vitamin A**	5,760 IU	10,000 IU
**Vitamin C**	600 mg	2,000 mg
**Vitamin D**	1,440 IU	2,000 IU
**Vitamin E**	360 IU	1,500 IU
**Vitamin B1**	18 mg	none set
**Vitamin B2**	13.5 mg	none set
^**a**^**Vitamin B3**	90 mg	35 mg
**Vitamin B5**	21.6 mg	none set
**Vitamin B6**	36 mg	100 mg
^**b**^**Folate**	1,440 mcg	1,000 mcg
**Vitamin B-12**	900 mcg	none set
**Vitamin H**	1,080 mcg	none set
**Calcium**	1,320 mg	2,500 mg
**Phosphorous**	840 mg	4,000 mg
^**c**^**Magnesium**	600 mg	350 mg
**Potassium**	240 mg	none set
**Iodine**	204 mcg	1,100 mcg
^**d**^**Zinc**	48 mg	40 mg
**Selenium**	204 mcg	400 mcg
**Copper**	7.2 mg	10 mg
**Manganese**	9.6 mg	11 mg
**Chromium**	624 mcg	none set
**Molybdenum**	144 mcg	2,000 mcg
**Iron**	13.74 mg	45 mg

Plus a proprietary formula of dl-phenylalanine, glutamine, citrus bioflavonoids, grape seed extract, choline bitartrate, inositol, ginkgo biloba, methionine, germanium sesquioxide, boron, vanadium, nickel.

For four ingredients, the amount in the full daily dose exceeds the tolerable upper intake levels (UL) set by the National Academy of Sciences:

^a ^The **B3 **(niacinamide) UL was set at 35 mg to prevent skin flushing, and is not based on a safety concern. The EMP+ B3 exceeds that UL, but there have been no reports of skin flushing problems in people taking this formula up to this point.

^b ^The **folate **UL was set at 1 mg to prevent masking a vitamin B12 deficiency. But of course, vitamin B12 deficiencies are unlikely to occur in someone taking this formula, because of the B12 content.

^c ^The **magnesium **UL of 350 mg was set because of concerns regarding diarrhea, but there do not appear to be any safety concerns.

^d ^The **zinc **UL is set at 40 mg, but the primary safety concern is that higher levels may result in an imbalance of copper. This is unlikely to occur in someone taking this formula, because of the copper content.

## Methods

### Data Sources

Medline was searched for empirical reports on EMP+ since the formula was developed in the late 1990s, and all investigators known by the authors and by the manufacturers were contacted. No report, published or unpublished, was excluded from this systematic review, and Additional File three contains all the data on EMP+ that is in existence up to the present time. Hence the data summarized here are not subject to reporting or publication bias, and there were no simplifying assumptions made that affected selection of data to be included. The data capture and preparation of the manuscript have followed PRISMA guidelines (refer to Additional Files 1 and 2 for the PRISMA checklist and flowchart).

The 36 ingredients of EMP+ are primarily vitamins and minerals. (All ingredients are listed on the developer's website (http://www.Truehope.com): 14 vitamins, 16 minerals, 3 amino acids, and 3 antioxidants.) There are currently 12 publications on EMP+ in the psychiatry and psychology literature, and other research is under review and in progress. So far research has emerged from three countries (Canada, New Zealand, the U.S.), involving scientists at multiple academic institutions, in addition to replications by clinicians in their private practices; none of the studies have been financially sponsored by the company. Although not yet studied in an RCT, the results of case-control designs, case studies using with-subject crossover designs, open-label case series, case reports with extensive historical treatment information, and two large database analyses are sufficiently promising to suggest that the formula may have some efficacy in the treatment of mood and anxiety symptoms in both adults and children [17-28].

Concerns about the potential for adverse effects are not equal across the ingredients contained in EMP+. For example, many of the nutrients are considered to be safe at up to 100 times the recommended nutrient intakes, because they are water soluble or because they are ubiquitous in our diet (Marks 1989): e.g., vitamins B1 (thiamine), B2 (riboflavin), B9 (folic acid), B12 (cobalamin), C (ascorbic acid), biotin, and pantothenic acid. In some cases such as riboflavin, it appears "that it is not possible to achieve a toxic dose by the oral route" [29]. In contrast, ingredients such as vitamin A (retinol), vitamin D (calciferols), vitamin B6 (pyridoxine), manganese, and vanadium bear closer scrutiny either because they are fat-soluble and stored in lipids, or because of insufficient existing information on safe doses for chronic ingestion.

When considering the safety and tolerability information on EMP+, the situation is complicated by the fact that the preparation has changed over time. Publications from 2001-4 used an older version that was often associated with digestive problems. At the end of 2002 the manufacturing process changed, most notably pulverizing the mineral particle size to <15 microns. The result decreased the formula's bulkiness, thereby requiring consumption of fewer capsules. Despite this physical change, the 36 ingredients have remained constant.

## Results

### Safety

Biological data on safety from 144 children and adults were available from six datasets (studies #1, 2, 3, 4, 7, 8 in Additional File 3). In these reports, there was not a single reported occurrence of a clinically meaningful negative outcome/effect or an abnormal blood test that could be attributed to toxicity.

The earliest pilot study (#8 in Additional File 3) was conducted by some of the present authors about 10 years ago with the earlier version of EMP+. Each of the 12 pediatric participants had a complete physical exam by the study physician prior to entering the trial, which was a within-subject crossover design. In each of the four-week segments, routine blood samples were collected, and heart rate and blood pressure were recorded. Although never submitted for publication, the results were described elsewhere [20]. An unpublished survey by the manufacturer (#7 in Additional File 3) resulted in the voluntary submission of blood test results by 27 adults that were reviewed and evaluated by a member of our team (JSAS). This information was requested to assist in assembling safety data for submission to a federal regulatory agency (Health Canada) in relation to academic research on the product. As with the first pilot study, these also were routine blood tests. Three other sources of safety data are studies from North America and New Zealand (#2-4 in Additional File 3), which are important for providing information on long-term exposure (>8 yrs) in both children and adults. In summary, based on these tests, no safety concerns emerged.

The most recent source of safety data (#1 in Additional File 3), reported here for the first time, is an RCT in medication-free adults with bipolar disorder carried out in two cities, one in Canada and the other in the United States. Randomization to 8 weeks of the active formula or placebo was followed by an 8-week open label extension. A full laboratory panel (Additional File 4) was completed at baseline, at the end of the randomization phase (Week 8) and at the end of the open label extension phase (Week 16). In addition, a smaller safety panel (hematology, potassium, calcium, alanine aminotransaminase, creatinine and estimated glomerular filtration rate (eGFR)) was performed every two weeks during each study phase. All laboratory results were reviewed on an ongoing basis by the lead psychiatrists at each site (JSAS and ETG) and also by the consulting nephrologist (EB). This study was approved by two Ethics Boards (one in Canada and one in the United States) but was terminated early for methodological and financial reasons; hence, it is informative for safety and tolerability, but not efficacy.

With corrections for multiple comparisons, no significant changes or group differences were noted from baseline screening to the end of the randomization phase or during the open label extension (Additional File 4). In addition, from the start of the open label extension at Week 8 to the end at Week 16, no group differences emerged for any variables. All values remained within normal clinical reference ranges throughout randomization and the open label.

### Tolerability

Adverse event (AE) information was available from 13 reports. Transient nausea and gastrointestinal discomfort were common with the previous version of EMP+ but are no longer a frequent occurrence, so the following results are from the 6 reports that employed the current formula (#1-6 in Additional File 3).

In the two reports by Rucklidge and colleagues (#2, #4 in Additional File 3) AEs were monitored in adults exposed to EMP+ for 8 weeks. In a recent case study [27], no adverse events occurred. In the case series of 14 adults with ADHD, headache was reported only in the first few weeks for four participants, nausea was reported by two people when consuming the formula on an empty stomach, and one participant had rash during the trial which the consulting psychiatrist reported was unrelated to the intervention as it had also occurred prior to exposure to EMP+.

The case-control study by Mehl-Madrona and colleagues (#3 in Additional File 3) provides unique AE data on the micronutrient formula in comparison to conventional medications [17]. Systematic monitoring of 22 physical signs and symptoms resulted in a report of 33 events affecting 19/44 patients receiving micronutrients, in comparison to 214 AEs from 44/44 patients receiving psychiatric medications. For 9 of the 22 signs and symptoms, individual chi square tests revealed significant group differences, with the micronutrient group always reporting fewer problems: increased appetite (p < .0001), fatigue (p < .0001), drowsiness (p < .0001), vomiting (p = 0.015), anxiety (p = 0.004), constipation (p = 0.026), dry mouth (p = 0.026), dyskinesia (p = 0.012), and akathisia (p = 0.026). In addition, the 44 children receiving micronutrients gained less weight (t(86) = -6.41, p < 0.0001), which is unlikely due to growth, as the length of follow-up time for the two groups did not differ.

Two other reports that commented on AEs included extensive case studies of young people with bipolar disorder (#5) and obsessive compulsive disorder (#6) who were successfully treated with EMP+. No safety data were provided, however the authors reported that AEs were monitored but none occurred.

Another source of adverse event data on the current version of EMP+ is the unpublished RCT described above (#1 in Additional File 3) in which adverse events were recorded at visits with a study psychiatrist (weekly during the randomization phase, and on four occasions during the open label extension). In total, 32 AEs were reported by 16 of 46 patients, none categorized by the study psychiatrist as being serious. The most commonly reported AEs (Additional File 3) were gastrointestinal problems such as nausea, vomiting, and diarrhea (46.9%), followed by headache (18.8%). The intensity of 87.5% of the AEs reported was mild or moderate, and 10% required adjustments in the dosage of the study formula. Four of the AEs (12.5%) were categorized as related to the study medication because the affected subjects had taken the formula on an empty stomach (contrary to instructions), and 16 (50%) of the AEs were categorized as possibly related to the study medication, although none resulted in patient withdrawal. The remaining AEs, most of which had also occurred prior to the study (e.g., eczema), were not thought to be related to the study medication. There was a significant association between the type of AE experienced and whether or not patients were on the active formula or the placebo (X^2^(5) = 11.91, p = .036): gastrointestinal problems occurred similarly in the two groups, but headaches were more common in the group receiving the active formula. During the conduct of this RCT, in which all subjects had confirmed Bipolar I or II (n = 46), two participants (both on active treatment) were withdrawn from randomization and moved into the open label phase when their symptoms became worse. One experienced worsening of a hypomanic phase and one of a depressive episode during the 8 weeks of treatment with EMP+, however both resolved during a further 8 weeks of active open label treatment.

## Discussion

The safety data presented here, derived from eight datasets, reveal the absence of clinically meaningful abnormal laboratory values. Similarly, the tolerability data, derived from six overlapping but non-identical datasets, amount to only minor, transitory adverse events, in particular headache and gastrointestinal problems. Given the significant and rapid growth in research on EMP+ as well as its use clinically around the world, the safety and tolerability data presented here are reassuring.

A significant concern of regulatory agencies is that mixtures of ingredients may have effects that are not seen with single ingredients either due to chemical interactions between the ingredients or to pharmacodynamic effects that are not obvious and cannot be studied ex vivo. The current compilation of available safety and tolerability data suggests that these effects are small with respect to EMP+.

There are many limitations to generalizing from these data to other complex formulae. The relationship between nutrition and toxicity is complex and we do not necessarily have a 'gold standard' to assess toxicity. Because of the growing popularity of alternative therapies in the mental health field, it would be interesting to compare EMP+ to conventional medication treatment. To date, only one study permitted such a direct assessment. In 44 patients taking micronutrients, matched in a case-control design with 44 treated with medication, all patients were reported to have normal laboratory values in repeated blood tests [17]. With respect to tolerability, by far the majority of AEs (214/246) were reported by the 44 children receiving conventional medication, who also had significant weight gain. Finally, we note that there is nothing in this review that evaluates the efficacy of treatment with either this or any other complex nutrient formula. No RCTs on this formula have yet been published.

Safety represents at least two different issues - safe with respect to general health or metabolic issues, and safe with respect to combining the formula with psychiatric medications. A significant limitation in the generalization of the results of this review is that only the first issue is addressed here. Given that mental health patients are heterogeneous with respect to genetic and metabolic profiles and that even the most well defined psychiatric conditions have no common known specific etiology, it is not surprising that addition of micronutrients to a pre-existing medication regimen is likely to be accompanied by complex interactions, which, if approached with insufficient caution, will result in unintended consequences. Based on the limited information in individuals treated with medication, we recommend that, not withstanding our findings of general safety of the formula when used in medication-free patients, use of multi-nutrient formulations as an adjunct should be monitored closely and with full attention to the possibility that optimum dosing of psychotropic agents may require significant adjustments. Recent data from a review of this issue revealed findings that are consistent with the interactions reported for EMP+: enhanced response to pharmaceuticals, especially in patients with depression and bipolar disorder [30].

## Conclusions

Although the safety and tolerability data reported here cannot be generalized to all complex micronutrient formulae, there are several reasons why it is particularly important to attend to the EMP+ reports. First, to the best of our knowledge, it has the largest number of ingredients (36) of any formula reported in the scientific literature. Second, there is more published and ongoing research on this formula for mental health than on any other complex formula anywhere in the world. Third, it is the formula for which research is primarily focused on mental disorders. And fourth, the hypothetical harm from consuming this formula has been an obstacle to scientists wanting to test its efficacy in specific mental health conditions.

## Competing interests

The authors declare that they have no competing interests.

## Authors' contributions

All authors have made substantial contributions to the conception and interpretation of the data presented here. JSAS, SGC, CF, and BJK drafted the manuscript and worked on successive revisions. ETG, CF, and EB were all involved in design and implementation of some of the data collection, as well as interpretation of the results. All authors read drafts near the completion of writing, and all have given final approval of the version submitted for publication.

## Pre-publication history

The pre-publication history for this paper can be accessed here:

http://www.biomedcentral.com/1471-244X/11/62/prepub

## Supplementary Material

Additional file 1 **PRISMA 2009 Checklist**. This file contains a completed PRISMA checklist. Click here for file

Additional file 2 **PRISMA flowchart**. This file contains a PRISMA flowchart for this Systematic Review. Click here for file

Additional file 3 **Studies with safety and tolerability information, in chronological order**. This file contains a table that lists all eight of the studies evaluated for this Systematic Review. Click here for file

Additional file 4 **Panels during the RCT**. This file contains a table that lists all of the laboratory results from participants who were in the randomized controlled trial. Click here for file
