# Supplementary material for: Systematic review of safety and tolerability of a complex micronutrient formula used in mental health
Source: BMC Psychiatry. 2011 Apr 18;11:62. doi: 10.1186/1471-244X-11-62 (PMC3094286; doi:10.1186/1471-244X-11-62)
Supplement: Additional file 2 — PRISMA flowchart. This file contains a PRISMA flowchart for this Systematic Review. [file 1471-244X-11-62-S2.DOC]

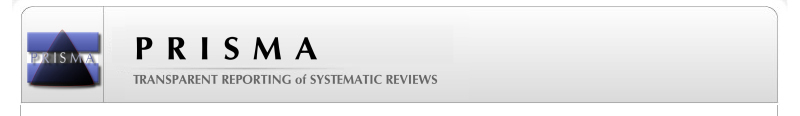
**PRISMA 2009 Flow Diagram**

**Screening**

**Included**

**Eligibility**

**Identification**

Records identified through database searching
(n = 0 )

Additional records identified through other sources
(n = 8 )

Records after duplicates removed
(n = 8 )

Records screened
(n = 8 )

Records excluded
(n = 0 )

Full-text articles assessed for eligibility
(n = 8 )

Full-text articles excluded, with reasons
(n = 0 )

Studies included in qualitative synthesis.
(n = 8 )

Studies included in quantitative synthesis (meta-analysis)
(n = 0 )
