# Supplementary material for: Systematic review of safety and tolerability of a complex micronutrient formula used in mental health
Source: BMC Psychiatry. 2011 Apr 18;11:62. doi: 10.1186/1471-244X-11-62 (PMC3094286; doi:10.1186/1471-244X-11-62)
Supplement: Additional file 3 — Studies with safety and tolerability information, in chronological order. This file contains a table that lists all eight of the studies evaluated for this Systematic Review. [file 1471-244X-11-62-S3.DOC]

Additional File 3. Studies with safety and tolerability information, in chronological order

| **Source** | **Sample** | **Duration of exposure** | **Variables** | **Clinically meaningful abnormal values** | **# AEs** | **Type of AE** | **Intensity of AE** | **Relationship of AE to EMP+** |
| --- | --- | --- | --- | --- | --- | --- | --- | --- |
| 1. Current report of RCT | 46 adults with Bipolar I or II, aged 18-63 yrs, medication-free, free of other chronic illnesses | 8 wks for the 23 patients randomized to placebo; 16 wks for the 23 randomized to active | Hematology, WBC diff., clinical chemistry, urinalysis | none | 32 in 16 patients | 46.9% were GI problems; 18.8% were headache | 87.5% were mild or moderate | 50% possibly related; 12.5% related |
| 1. Rucklidge & Harrison, 2010 [27] | Case study of an adult with Bipolar II and ADHD, aged 21 yrs | 8 wks | Hematology and biochemistry including thyroid function, serum lipids, prolactin and glucose, blood clotting, urinalysis | none | 0 | n/a | n/a | n/a |
| 1. Mehl-Madrona et al., 2010 [17] | 44 children and young adults diagnosed with autism spectrum disorder, aged 28 yrs | 3-98 months | BP, hematology, WBC diff., clinical chemistry, urinalysis | none | 33 in 44 patients (compared to 214 in the 44 patients treated with conventional medications) | Most frequent was stomach ache (9 of the 33) | Not reported | Not reported |
| 1. Rucklidge et al., 2010 [26] | 14 adults with ADHD and severe mood dysregulation, aged 18-55 yrs | 8 wks | Hematology and biochemistry including thyroid function, serum lipids, prolactin and glucose, blood clotting, urinalysis | none | 7 in 14 patients | Headache (n=4; 28.6%), nausea (n=2;14.3%), rash (n=1;7.1%) | Not reported | Headache and nausea possibly related; rash not related |
| **Source** | **Sample** | **Duration of exposure** | **Variables** | **Clinically meaningful abnormal values** | **# AEs** | **Type of AE** | **Intensity of AE** | **Relationship of AE to EMP+** |
| 1. Frazier et al., 2009 [25] | 1 child with bipolar disorder, psychotic features, GAD, OCD | 14 mos. | n/a | n/a | None reported | n/a | n/a | n/a |
| 1. Rucklidge, 2009 [28] | 1 young adult with OCD, major depression, Asperger’s | 8 wks, followed by a break; then 6 mos | n/a | n/a | none | n/a | n/a | n/a |
| 1. Unpublished survey data, submitted to Health Canada (2004) | 27 adults aged 20-74 yrs | From 10-41 months | Hematology, WBC diff., clinical chemistry, urinalysis^a^ | none | n/a | n/a | n/a | n/a |
| 1. Unpublished pilot data (described in Kaplan et al., 2004) [20] | 12 children with mood and anxiety symptoms, aged 8-16 yrs | Two periods of 4 wks each | HR, BP, physical exam, hematology, WBC diff., clinical chemistry, urinalysis | none | n/a | n/a | n/a | n/a |

Studies with safety and tolerability information, in chronological order (continued)

^a^ Urinalyses were available for only 16 participants

Abbreviations: GAD = generalized anxiety disorder, OCD = obsessive compulsive disorder, HR = heart rate, BP = blood pressure, WBC diff = white blood cell differential, n/a = not applicable
