# Supplementary material for: Systematic review of safety and tolerability of a complex micronutrient formula used in mental health
Source: BMC Psychiatry. 2011 Apr 18;11:62. doi: 10.1186/1471-244X-11-62 (PMC3094286; doi:10.1186/1471-244X-11-62)
Supplement: Additional file 4 — Panels during the RCT. This file contains a table that lists all of the laboratory results from participants who were in the randomized controlled trial. [file 1471-244X-11-62-S4.DOC]

Additional File 4. Panels during the RCT

|  | Baseline Screening | | | | End of Randomization | | | | End of Open Label | | | |
| --- | --- | --- | --- | --- | --- | --- | --- | --- | --- | --- | --- | --- |
|  | Placebo | | Active | | Placebo | | Active | | Placebo | | Active | |
|  | Mean or Freq | SD | Mean or Freq | SD | Mean or Freq | SD | Mean or Freq | SD | Mean or Freq | SD | Mean or Freq | SD |
| Hemoglobin | 145.95 | 11.86 | 144.09 | 13.84 | 145.74 | 11.59 | 143.91 | 12.98 | 143.18 | 10.51 | 141.29 | 13.50 |
| Hematocrit | 0.42 | 0.03 | 0.42 | 0.04 | 0.42 | 0.03 | 0.42 | 0.03 | 0.42 | 0.03 | 0.41 | 0.04 |
| RBC | 4.72 | 0.39 | 4.79 | 0.43 | 4.74 | 0.35 | 4.76 | 0.42 | 4.67 | 0.34 | 4.89 | 0.69 |
| MCV | 90.12 | 3.20 | 87.90 | 4.23 | 89.45 | 2.68 | 87.98 | 4.48 | 90.48 | 4.67 | 88.12 | 4.80 |
| MCHC | 343.60 | 4.98 | 342.45 | 6.11 | 343.63 | 5.33 | 343.09 | 5.34 | 343.89 | 3.82 | 343.17 | 4.37 |
| RDW | 13.30 | 0.60 | 12.96 | 0.69 | 13.24 | 0.67 | 13.39 | 1.06 | 13.28 | 0.73 | 13.45 | 0.92 |
| Platelet count | 267.84 | 40.15 | 258.09 | 69.26 | 266.68 | 48.46 | 254.26 | 64.66 | 265.27 | 36.29 | 260.65 | 59.94 |
| WBC | 5.94 | 1.02 | 6.40 | 1.56 | 6.34 | 1.85 | 6.02 | 1.25 | 6.39 | 1.73 | 6.08 | 1.13 |
| Neutrophils | 3.35 | 1.11 | 3.54 | 1.19 | 3.41 | 1.80 | 3.22 | 1.33 | 3.24 | 1.71 | 2.93 | 1.44 |
| Lymphocytes | 1.70 | 0.62 | 1.89 | 0.72 | 1.74 | 0.82 | 1.69 | 0.75 | 1.82 | 0.67 | 1.85 | 1.12 |
| Monocytes | 0.41 | 0.16 | 0.42 | 0.16 | 0.41 | 0.24 | 0.38 | 0.16 | 0.42 | 0.18 | 0.37 | 0.21 |
| Eosinophils | 0.13 | 0.11 | 0.12 | 0.07 | 0.12 | 0.10 | 0.14 | 0.14 | 0.12 | 0.09 | 0.10 | 0.08 |
| Basophils | 0.01 | 0.03 | 0.01 | 0.03 | 0.01 | 0.03 | 0.00 | 0.00 | 0.00 | 0.00 | 0.01 | 0.03 |
| INR | 1.01 | 0.08 | 1.02 | 0.05 | 1.01 | 0.06 | 1.02 | 0.06 | 1.00 | 0.05 | 1.13 | 0.37 |
| PTT | 27.96 | 7.89 | 30.00 | 6.65 | 27.92 | 7.99 | 30.16 | 6.79 | 27.73 | 10.12 | 30.38 | 9.62 |
| Glucose fasting | 4.95 | 0.49 | 4.96 | 0.44 | 5.04 | 0.52 | 4.97 | 0.53 | 5.20 | 0.47 | 5.17 | 0.63 |
| Sodium | 140.90 | 2.19 | 139.95 | 2.13 | 140.90 | 2.27 | 139.41 | 1.68 | 125.67 | 43.41 | 138.92 | 2.02 |
| Potassium | 4.32 | 0.39 | 4.20 | 0.33 | 4.32 | 0.42 | 4.17 | 0.33 | 4.38 | 0.37 | 4.12 | 0.61 |
| Chloride | 105.43 | 2.64 | 104.73 | 1.96 | 105.30 | 2.66 | 103.64 | 3.13 | 104.44 | 3.61 | 94.53 | 29.59 |
| CO2 content | 24.29 | 2.35 | 24.32 | 2.46 | 24.65 | 2.56 | 24.00 | 2.71 | 26.11 | 2.32 | 25.58 | 3.48 |
| Alkaline phosphatase | 63.67 | 13.91 | 71.81 | 16.77 | 64.95 | 14.97 | 69.59 | 18.16 | 60.78 | 16.40 | 62.42 | 17.43 |
| ALT | 17.86 | 5.45 | 26.41 | 18.73 | 18.35 | 5.92 | 24.87 | 18.05 | 22.73 | 6.92 | 25.18 | 13.03 |
| AST | 19.95 | 4.04 | 23.67 | 11.16 | 20.70 | 3.85 | 24.23 | 10.89 | 23.44 | 4.39 | 22.58 | 6.37 |
| Bilirubin, total | 9.99 | 2.46 | 8.35 | 3.18 | 8.93 | 3.57 | 8.21 | 3.66 | 8.41 | 2.84 | 7.44 | 2.52 |
| Calcium | 2.35 | 0.06 | 2.34 | 0.11 | 2.36 | 0.06 | 2.34 | 0.09 | 2.35 | 0.09 | 2.34 | 0.07 |
| CK | 95.24 | 55.28 | 95.00 | 42.70 | 108.90 | 59.96 | 95.95 | 46.86 | 106.00 | 87.67 | 81.67 | 36.81 |
| Creatinine | 73.71 | 14.73 | 71.29 | 11.58 | 74.32 | 14.18 | 73.03 | 10.68 | 75.66 | 13.39 | 73.70 | 10.22 |
| Estimated GFR  (ml/min/1.73 sq. m.) | all > 60 | | all > 60 | | all > 60 | | all > 60 | | all > 60 | | all > 60 | |

| Panels during the RCT (continued) | Baseline Screening | | | | End of Randomization | | | | End of Open Label | | | |
| --- | --- | --- | --- | --- | --- | --- | --- | --- | --- | --- | --- | --- |
|  | Placebo | | Active | | Placebo | | Active | | Placebo | | Active | |
|  | Mean or Freq | SD | Mean or Freq | SD | Mean or Freq | SD | Mean or Freq | SD | Mean or Freq | SD | Mean or Freq | SD |
| Urea | 4.65 | 1.39 | 5.13 | 1.40 | 4.69 | 2.17 | 5.13 | 1.49 | 4.91 | 1.23 | 4.23 | 0.96 |
| GGT | 16.57 | 6.45 | 23.19 | 13.71 | 17.00 | 6.67 | 21.91 | 12.91 | 20.00 | 8.03 | 24.83 | 13.83 |
| Phosphate | 1.09 | 0.14 | 1.11 | 0.16 | 1.09 | 0.16 | 1.11 | 0.17 | 0.99 | 0.10 | 1.07 | 0.21 |
| Protein, total | 71.33 | 3.67 | 72.57 | 4.66 | 71.60 | 3.55 | 72.18 | 4.18 | 70.56 | 4.50 | 73.00 | 2.66 |
| Albumin | 43.00 | 2.58 | 42.36 | 3.90 | 43.05 | 2.82 | 42.41 | 3.61 | 41.22 | 2.28 | 41.25 | 3.44 |
| Urate | 322.57 | 65.36 | 319.75 | 76.30 | 313.36 | 60.33 | 307.65 | 78.05 | 280.00 | 68.21 | 296.78 | 104.68 |
| Cholesterol | 4.68 | 1.13 | 4.67 | 1.05 | 4.66 | 1.02 | 4.76 | 1.11 | 4.98 | 1.22 | 5.13 | 1.23 |
| TSH | 1.91 | 1.50 | 2.03 | 0.93 | 1.90 | 1.46 | 2.28 | 1.27 | 2.28 | 1.54 | 2.28 | 1.16 |
| Ceruloplasmin | 0.26 | 0.08 | 0.24 | 0.08 |  |  |  |  |  |  |  |  |
| Urinalysis color normal | 100% |  | 100% |  | 100% |  | 100% |  | 100% |  | 100% |  |
| Appearance normal | 100% |  | 100% |  | 100% |  | 100% |  | 100% |  | 100% |  |
| Specific gravity normal | 100% |  | 100% |  | 100% |  | 100% |  | 100% |  | 100% |  |
| pH | 6.53 | 0.70 | 6.39 | 0.76 | 6.70 | 0.59 | 6.46 | 0.82 | 6.94 | 0.82 | 6.89 | 0.60 |
| Leukocyte absent | 88.2% |  | 94.7% |  | 84.6% |  | 86.7% |  | 100% |  | 100% |  |
| Nitrite absent | 100% |  | 100% |  | 100% |  | 100% |  | 100% |  | 100% |  |
| Protein absent | 94% |  | 100% |  | 93.3% |  | 93.3% |  | 100% |  | 100% |  |
| Glucose absent | 100% |  | 100% |  | 100% |  | 100% |  | 100% |  | 100% |  |
| Ketones absent | 100% |  | 100% |  | 100% |  | 100% |  | 100% |  | 100% |  |
| Blood absent | 83.3% |  | 89.5% |  | 86.7% |  | 100% |  | 100% |  | 100% |  |
| Microscopic WBC absent | 90.4% |  | 95.8% |  | 90.4% |  | 95.8% |  | 100% |  | 95.8% |  |
| Microscopic RBC absent | 95.2% |  | 95.8% |  | 95.2% |  | 100% |  | 100% |  | 100% |  |
| Urine drug screen positive | 0% |  | 0% |  | 0% |  | 0% |  | 0% |  | 0% |  |
| Pregnancy test positive | 0% |  | 0% |  | 0% |  | 0% |  | 0% |  | 0% |  |
